# Change of Dietary and Lifestyle Habits during and after the COVID-19 Lockdown in Cyprus: An Analysis of Two Observational Studies

**DOI:** 10.3390/foods11141994

**Published:** 2022-07-06

**Authors:** Maria Kyprianidou, Stavri Chrysostomou, Costas A. Christophi, Konstantinos Giannakou

**Affiliations:** 1 Department of Health Sciences, School of Sciences, European University Cyprus, Nicosia 1516, Cyprus; kyprianidou.maria.ky@gmail.com; 2 Department of Life Sciences, School of Sciences, European University Cyprus, Nicosia 1516, Cyprus; s.chrysostomou@euc.ac.cy; 3 Cyprus International Institute for Environmental and Public Health, Cyprus University of Technology, Limassol 3036, Cyprus; costas.christophi@cut.ac.cy

**Keywords:** diet, lifestyle habits, coronavirus, COVID-19, nutrition, quarantine, lockdown, Cyprus

## Abstract

Background: People’s dietary and lifestyle habits appeared to be influenced by restrictive measures imposed in response to the COVID-19 pandemic. This study examines the differences in dietary and lifestyle habits during and after the lockdown measures in Cyprus. Methods: Two online cross-sectional surveys were conducted, using a self-administered, anonymous questionnaire to collect information on sociodemographic and anthropometric characteristics, smoking habits, physical activity, and dietary habits. The first survey was conducted between 6 April 2020 and 20 June 2020 (during national lockdown) while the second survey was conducted between 27 October 2021 and 20 January 2022 (post-lockdown). Results: A total of 2503 individuals participated in the study. A higher consumption of fruits, vegetables, legumes/pulses, fish, and poultry was identified during lockdown compared to the period after the lockdown. Moreover, a greater daily intake of olive oil and a lower consumption of alcohol was found during the confinement period compared to the post-confinement period. During lockdown, most participants (43.0%) never or rarely used delivery services, while the largest proportion of the participants after lockdown used delivery services 1–3 times per month (37.0%) (*p* < 0.001). During lockdown, around 66% of the participants were physically active, compared to 55.5% after lockdown (*p* < 0.001). Furthermore, when compared to those with a normal BMI, more overweight and obese respondents ordered food 1–2 times per week in both periods (*p* < 0.001). Conclusions: Dietary and lifestyle habits of the participants were healthier throughout the lockdown period than after the end of the restrictive measures due to the COVID-19 pandemic. It is critical to encourage the Cypriot population to maintain the healthy dietary and lifestyle habits established during the lockdown in their daily lives after the confinement.

## 1. Introduction

SARS-CoV-2, also known as coronavirus disease 2019 (COVID-19) is a severe acute respiratory syndrome first reported in Wuhan, China [1]. Since March 2020, the World Health Organization (WHO) has declared it a global pandemic [2], prompting public health authorities around the world to implement lockdown measures as a pandemic-prevention approach [3,4,5]. The amount of hours allowed for outdoor activities were limited due to the restrictive measures, whilst the closure of restaurants, cafes, schools, universities and sports facilities, as well as online working and online learning, also took place [4].

Certain life-altering experiences, such as the COVID-19 pandemic, may influence an individual’s daily routine for better or worse. It is true that when daily habits and environment change, eating behavior may change too [6]. Therefore, worldwide, authorities and health care professionals recommended staying healthy during the pandemic by eating plenty of fresh fruits and vegetables, reducing stress, and staying active [7]. To date, many studies have investigated the effects of lockdown due to the COVID-19 pandemic on changes in dietary habits [3,5,8,9,10,11,12,13,14,15,16]. In fact, a recent scoping review investigated the impact of lockdown on dietary habits in various population groups and showed that a lockdown could both negatively and positively influence dietary habits [17]. Negative dietary changes may occur because of low access to fresh food [18], while positive dietary changes could be attributed to increased time for cooking and limited access to delivery services [17]. Recent studies in Cyprus [14] and Spain [10] found a greater adherence to the Mediterranean diet during the lockdown period, with an increase in the consumption of fruits and vegetables. Moreover, an online observational study which examined dietary habits before and during the COVID-19 pandemic in Poland, Austria, and the United Kingdom found that dietary habits changed during that period and contributed to an increased body weight, which in turn could have several health-threatening consequences [16]. Moreover, in another study, it was shown that the majority of Italians did not follow healthy dietary habits during the pandemic [17]. Other studies found an improvement in the quality of diet among young individuals [19,20,21], where increased cooking frequency was found to be associated with improved diet quality [21]. For instance, a study that investigated the change in dietary habits in the Spanish population after confinement reported that younger individuals spent less time preparing meals than older individuals [21]. During the COVID-19 lockdown period, there was a higher adherence to the Mediterranean diet (which is a healthy diet) among Italians aged 18–30 years old compared to the elderly and younger population [20], as well as among Croatians aged 20–50 years old [18]. In contrast, another study conducted in Italy with young adults (mean age = 29.8 years old) discovered that more than half of the participants had a low adherence to the Mediterranean diet and a higher consumption of foods high in added sugars and saturated fats [17].

Notably, lifestyle habits, including physical activity and smoking status, also changed during the lockdown period. In particular, due to the imposition of many lockdowns and quarantines in several countries, social distancing, and compulsory home isolation in response to the COVID-19 pandemic, sedentary lifestyle had increased [22]. Moreover, the interruption of the daily (work) routine caused by staying at home (digital education, working from home) resulted in boredom and lower physical activity levels [23]. Furthermore, various changes in physical activity patterns have been recorded [24,25,26] as a result of gyms and sports facilities being closed during lockdowns, and physical activity at home was sometimes unavailable due to a shortage of space. Indeed, the positive results of the restrictive measures, quarantines, and lockdowns potentially reduced the transmission of the virus but also reduced the levels of physical activity in the global population [26,27]. In addition, increased stress and the economic crisis due to the pandemic led to changes in other lifestyle habits, including smoking behaviors. In particular, research has shown an increased prevalence of smoking [27,28]. In contrast, a recent cross-sectional study found that the COVID-19 outbreak has resulted in unexpected changes in smoking habits, with an equal percentage saying they had increased or decreased their smoking [29]. Therefore, findings related to lifestyle changes seem to be controversial and further research is needed to investigate these changes.

Due to the inconsistency in research findings regarding the effects of COVID-19 on dietary and lifestyle habits, it is necessary to examine the changes in health-related behaviors such as dietary habits, physical activity level, and smoking habits during and after COVID-19 lockdown at a population level. Governments’ policy responses to reduce the spread of COVID-19 differed across countries. A stringency index is a composite measure based on nine response indicators, including school closures, workplace closures, and travel bans, which records the strictness of government policies (range 0–100, with higher values indicating stringent measures) [30,31]. In Cyprus, the stringency index from 6 April 2020 to 20 June 2020 was 84.9, while the stringency index from 27 October 2021 to 20 January 2022 was 54.1 [31]. The assessment of possible changes in health-related behaviors among the population of Cyprus during and after the period of the COVID-19 lockdown could potentially provide valuable knowledge. Thus, this study aims to examine the differences in dietary habits (consumption of selected foods and general food habits), lifestyle habits (physical activity level and smoking habits), and physical characteristics (obesity status) among the adult population of Cyprus during and after COVID-19 lockdown restrictive measures.

## 2. Materials and Methods

### 2.1. Study Design, Participants, and Procedure

This analysis comprised two cross-sectional online surveys. The first cross-sectional survey was conducted from 6 April 2020 until 20 June 2020, when the first wave of the COVID-19 pandemic occurred and a national lockdown was implemented in Cyprus. During this period, almost all outdoor activities (such as mass gatherings and events) were banned, and the population of Cyprus was forced to quarantine at home. Individuals could leave their home only for essentials, such as visiting food markets and pharmacies, and going to work (when being physically present at the workplace was essential). The second cross-sectional survey was conducted between 27 October 2021 and 20 January 2022, when strict restrictive measures due to the COVID-19 pandemic were no longer applicable. Citizens only presented their safe pass when visiting shopping centers, supermarkets, restaurants, and entertainment centers (i.e., cinemas and theatres).

The study sample included Greek Cypriot-speaking men and women aged 18 years old and over, living in the five government-controlled districts of the Republic of Cyprus (Nicosia, Limassol, Larnaca, Paphos, and Ammochostos). A nonprobability convenience sampling approach was used to recruit participants, using an online self-administered questionnaire created in Google Forms and dispersed using instant messaging apps (e.g., Facebook Messenger, WhatsApp, Viber), social media platforms (e.g., Facebook, Instagram), and social networking sites (e.g., LinkedIn).

### 2.2. Measurements

A self-administered questionnaire was used to collect information on sociodemographic and anthropometric characteristics, consumption of selected foods and general food habits, physical activity, and smoking habits.

#### 2.2.1. Sociodemographic Characteristics

Age was reported in years and gender was recorded as male or female. Geographical area was categorized as one of the government-controlled districts of the Republic of Cyprus, while residency was recorded as urban or rural. Marital status was recorded as married/in cohabitation, unmarried, or divorced/separated/widowed. Having children was evaluated using a binary question (Yes vs. No). Educational level was classified into three categories: (i) primary education (participants who completed only primary school—less than 7 years of schooling); (ii) secondary education (participants who completed middle or high school—7 to 12 years of schooling); (iii) higher education (participants who have a university degree—more than 12 years of schooling). Annual income was classified as low (less than 6500 euros), moderate (6500–19,500 euros), and high (more than 19,500 euros).

#### 2.2.2. Anthropometric Characteristics

Weight and height were self-reported in kilograms and in meters, respectively. BMI was calculated as weight divided by height squared. According to the WHO classification [32], obesity was defined as BMI > 29.9 kg/m^2^, overweight as BMI 25–29.9 kg/m^2^, normal as BMI 18.5–24.9 kg/m^2^, and underweight as BMI < 18.5 kg/m^2^.

#### 2.2.3. Smoking Habits and Physical Activity Assessment

Smoking habits were evaluated using a question on smoking status (i.e., non-smoker, current smoker, and past smoker). Physical activity was evaluated using a binary question (not adequately physically active vs. physically active), whilst type of exercise was categorized as football, volleyball, basketball, swimming, martial arts, gym, track, jogging, and cycling, and there was an option to report another type of exercise. Duration of exercise per week was recorded as less than one hour, 1–3 h, 3–6 h, 6–9 h, and more than 9 h.

#### 2.2.4. Dietary Habits Assessment

Dietary assessment was evaluated using a validated questionnaire developed by Panagiotakos et al. [33]. The questionnaire collects information on the consumption of 11 food groups: (a) non-refined cereals, (b) fruits, (c) vegetables, (d) legumes/pulses, (e) potatoes, (f) fish, (g) meat and meat products, (h) poultry, (i) full-fat dairy products, (j) olive oil, and (k) alcohol intake [any type of alcohol consumption was measured in wine glasses (100 mL) and quantified by ethanol intake (grams/day)].

The frequency of participants’ use of delivery services was also recorded (never/rarely, 1–3 times/month, 1–2 times/week, 3–6 times/week, 1 time/day, 2 times/day). Coffees per day was classified as 0, 1, 2, 3, 4, or more than 4 coffees per day. In addition, two questions were used to identify the type of milk and sugar used in coffee. Type of milk was categorized as no milk, full-fat, low fat (1.5%), no fat (0%), lactose-free, soya, almond, and coconut, and there was an option to report another type of milk. Type of sugar was categorized as no sugar, white, brown, stevia, and sweetener, and there was an option to report any other type of sugar. Dietary supplements were reported using the question “If you are taking dietary supplements, please specify which dietary supplements you are taking,” to which participants could respond as they wished, with possible answers including vitamin C, vitamin D, multivitamins, calcium, magnesium, proteins, creatine, and the option “other”.

### 2.3. Ethics Approval

Both surveys were approved by the Cyprus National Bioethics Committee (CNBC) (ΕΕΒΚ ΕΠ 2020.01.57, ΕΕΒΚ ΕΠ 2021.01.96). Participation was completely anonymous and voluntary, and participants consented to take part in the research before completing the online questionnaire by answering a “Yes/No” question on a mandatory electronic form.

### 2.4. Statistical Analysis

The distribution of the continuous variables was examined using the Shapiro–Wilk normality test. Participants’ characteristics are presented as mean ± standard deviation (SD) for continuous measures and absolute (*n*) and relative (%) frequencies for categorical variables. To assess the differences in categorical dietary habits and lifestyle characteristics among the first cross-sectional survey (during lockdown) and the second cross-sectional survey (post-lockdown), the chi-square test of independence was used. The Student’s t-test was used for the comparison of continuous dietary habits and lifestyle characteristics. Dietary habits among the first cross-sectional study and the second cross-sectional study for females and males, as well as among the BMI categories, were separately assessed using the chi-square test of independence. Bar charts were constructed to present the type of exercise and dietary supplements. All statistical tests performed were two-sided, with the statistical significance level set at α = 0.05. Statistical analysis was conducted using STATA 14.0 (Stata Corp, College Station, TX, USA) [34] and Microsoft Excel 2013.

## 3. Results

Due to the limited number of participants >65 years old, the age threshold was set to less than 65 years old in order to have comparable samples. 1485 adults participated in the first survey. Among them, men and women aged less than 65 years old (*n* = 1460) were selected for the purposes of this analysis. In addition, 1045 adults participated in the second survey, and men and women aged less than 65 years old (*n* = 1043) were also selected.

### 3.1. Sociodemographic Characteristics

There were a total of 2503 participants, of which 1460 (58.3%) were from the first survey and 1043 (41.7%) from the second survey. The mean age of the individuals from the first and the second surveys was 35.3 years old (SD = 11.4 years) and 32.2 years old (SD = 11.1 years), respectively (*p* < 0.001) (Table 1). Overall, the majority of the respondents were female (*n* = 1594, 64.0%), lived in the capital of Cyprus, Nicosia (*n* = 1372, 55.0%), and were residents of urban areas (*n* = 2044, 82.0%). In addition, most of the participants were married/in cohabitation (*n* = 1201, 48.4%), without children (*n* = 1414, 56.8%), had completed a form of higher education (*n* = 1938, 77.5%), and had an annual income of more than €19,500 euros (*n* = 900, 36.1%). In particular, during lockdown, most of the participants were female (*n* = 875, 60.5%), lived in Nicosia (*n* = 779, 53.7%), were residents of urban areas (*n* = 1208, 83.3%), were married/in cohabitation (*n* = 746, 51.6%), without children (*n* = 765, 52.7%), had completed a form of higher education (*n* = 1205, 82.7%), and had an annual income of more than €19,500 euros (*n* = 645, 44.3%). After lockdown, most of the respondents were female (*n* = 719, 68.9%), lived in Nicosia (*n* = 593, 56.9%), were residents of urban areas (*n* = 836, 80.2%), were unmarried (*n* = 538, 51.9%), without children (*n* = 649, 62.6%), had completed a form of higher education (*n* = 733, 70.3%), and had an annual income from €6500 to €19,500 euros (*n* = 551, 53.2%). More information regarding the sociodemographic characteristics of participants in the first cross-sectional survey and the second cross-sectional survey is presented in Table 1.

### 3.2. Dietary Habits

#### 3.2.1. Food Consumption in Total Samples and by Gender

Table 2 presents the results of the food consumption survey in total samples and by gender. During lockdown, 466 (32.0%) participants reported that they consumed 1–4 portions of fruits per week and 395 (27.1%) 5–8 portions per week, while the corresponding percentages among the individuals after lockdown were 35.1% (*n* = 366) and 26.0% (*n* = 271), respectively (*p* = 0.042). We also found statistically significant differences for consumption of vegetables (*p* < 0.001) and legumes/pulses (*p* < 0.001) during and after lockdown. Specifically, a large proportion of the respondents consumed 1–6 portions (34.9% and 36.7%, during and after lockdown, respectively) and 7–12 portions (31.9% and 27.4%, during and after lockdown, respectively) of vegetables per week (*p* < 0.001). In addition, half of the participants consumed 1–2 portions of legumes/pulses per week during lockdown (*n* = 730, 50.0%), followed by the consumption of 3–4 portions of legumes/pulses per week (*n* = 378, 25.9%), while the participants after the COVID-19 lockdown consumed 1–2 portions of legumes/pulses per week (*n* = 510, 49.1%) followed by the consumption of less than 1 portion of legumes/pulses per week (*n* = 222, 21.4%) (*p* < 0.001).

Statistically significant differences between the consumption of fish (*p* < 0.001), poultry (*p* < 0.001), and full-fat dairy products (*p* = 0.004) during and post-lockdown were found. The largest percentages of the individuals who participated in the first survey consumed 1–2 portions of fish per week (*n* = 670, 45.9%), 3 or less than 3 portions of poultry per week (*n* = 812, 55.9%), and 10 or less than 10 portions of full-fat dairy products per week (*n* = 994, 68.3%), while the largest percentages of the individuals who participated in the study after lockdown consumed less than 1 portion of fish per week (*n* = 399, 38.3%), 3 or less than 3 portions of poultry per week (*n* = 469, 45.5%), and 10 or less than 10 portions of full-fat dairy products per week (*n* = 669, 64.5%). During the COVID-19 lockdown, we identified statistically significant differences for the consumption of potatoes (*p* = 0.020), meat and meat products (*p* < 0.001), poultry (*p* < 0.001), olive oil (*p* = 0.009) and alcohol intake (*p* < 0.001) between females and males. The largest differences in the consumption of meat and meat products between females and males were identified among those who consumed one or less than 1 portion per week and those who consumed more than 10 portions per week (*p* < 0.001). Post-lockdown, we identified statistically significant differences for the consumption of non-refined cereals (*p* = 0.026), potatoes (*p* < 0.001), meat and meat products (*p* < 0.001), poultry (*p* < 0.001), and full-fat dairy products (*p* < 0.001) between females and males. In addition, a considerably higher proportion of daily consumption of olive oil (*n* = 612, 42.0%) was identified during lockdown compared to the proportion post-lockdown (*n* = 313, 30.1%) (*p* < 0.001).

#### 3.2.2. Olive Oil and Alcohol Consumption

A considerably higher proportion of daily consumption of olive oil (*n* = 612, 42.0%) as well as the consumption of less than 300 mL alcohol (*n* = 1309, 91.1%) were identified during lockdown, compared to the proportions post-lockdown (*n* = 313, 30.1% and *n* = 891, 86.8%, for olive oil consumption and alcohol intake, respectively) (*p* < 0.001). Moreover, 72.2% (*n* = 13) of individuals who never used olive oil during lockdown, as well as 64.0% (*n* = 388) of the respondents who used olive oil daily, were female (*p* < 0.001). In addition, the largest differences between females and males for olive oil consumption after the lockdown period were identified among those who consumed olive oil never (*n* = 10, 71.4% and *n* = 4, 28.6%, for females and males, respectively), rarely (*n* = 91, 76.5% and *n* = 28, 23.5%, for females and males, respectively), or daily (*n* = 239, 76.4% and *n* = 74, 23.6%, for females and males, respectively) (*p* = 0.001) (Supplementary Table S1).

#### 3.2.3. Use of Delivery Services

Most of the participants used delivery services never or rarely during lockdown (*n* = 626, 43.0%), while most participants post-lockdown used delivery services 1–3 times per month (*n* = 385, 37.0%) (*p* < 0.001). Among those who used delivery services never or rarely during the lockdown period, the largest proportion was female (*n* = 418, 67.5%) (*p* < 0.001) (Supplementary Table S2).

#### 3.2.4. Coffee Drinking in Total Samples and by Gender

A considerably higher proportion of the consumption of more than 4 coffees per day was identified in males compared to females during (*p* = 0.013) and after lockdown (*p* < 0.001). We reported statistically significant differences among types of milk and sugar during and after lockdown (*p* < 0.001) (Supplementary Table S2).

#### 3.2.5. Use of Dietary Supplements in Total Samples

**Figure 1:**
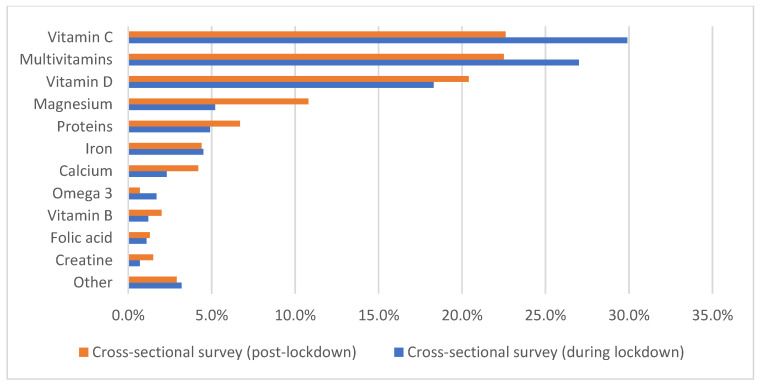
Use of dietary supplements among the first cross-sectional survey (during lockdown) and the second cross-sectional survey (post-lockdown). Note: Vitamin B included Vitamin B12, Vitamin B1, Vitamin B, and B-complex; “other” included probiotics, echinacea, zinc, ferritin, cod liver oil, glucosamine/chondroitin, spirulina, curcumin, cranberries, fish oil, amino acids, vitamin E, turmeric, and iodine.

The most common dietary supplement during and after lockdown periods was vitamin C (29.9% and 22.6% during and after lockdown, respectively), followed by multivitamins (27.0% and 22.5% during and after lockdown, respectively), and vitamin D (18.3% and 20.4% during and after lockdown, respectively) (Figure 1).

### 3.3. Smoking Habits and Physical Activity

We found that 65.1% of the individuals who participated in the study during lockdown (*n* = 949) were non-smokers, while the corresponding percentage among the individuals participating in the study after lockdown (*n* = 686) was 66.0% (*p* < 0.001) (Table 3). Furthermore, almost 66% (*n* = 959) of the study participants during lockdown were physically active, while among the study participants after lockdown the corresponding proportion was 55.5% (*n* = 578) (*p* < 0.001).

**Figure 2:**
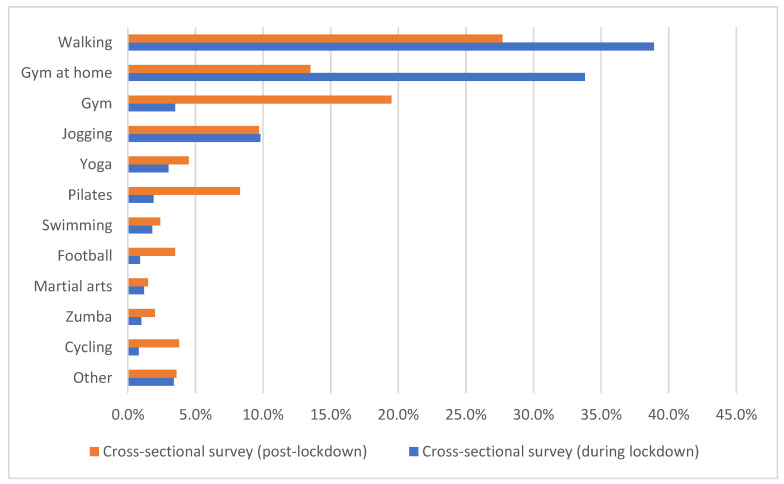
Type of exercise among first cross-sectional survey (during lockdown) and the second cross-sectional survey (post-lockdown). Note: “Other” included CrossFit/TRX, handball, aerobics, volleyball, bodybuilding, functional training, calisthenics, tennis, shooting, rhythmic gymnastics, track, and sailing/rowing.

The most common type of exercise in both time periods was walking (38.9% and 27.7% during and post-lockdown, respectively) (Figure 2). In addition, during lockdown, gym at home (33.8%) and jogging (9.8%) were among the most common types of exercise, whereas after lockdown, these were gym (19.5%) and gym at home (13.5%).

### 3.4. Food Consumption by BMI Category

The mean BMI of the participants during lockdown was 25.1 kg/m^2^ (SD = 4.9 kg/m^2^), while the mean BMI post-lockdown was 24.3 kg/m^2^ (SD = 5.1 kg/m^2^) (*p* < 0.001). More specifically, during lockdown about half of the participants (*n* = 736, 50.9%) had a normal weight, 28.4% (*n* = 411) were overweight, 15.8% (*n* = 228) were obese, and almost 5% (*n* = 71) were underweight, while the corresponding percentages among the respondents who participated in the second survey were 56.4% (*n* = 584), 25.2% (*n* = 261), 12.2% (*n* = 127), and 6.2% (*n* = 64) (*p* < 0.001), respectively (Table 3).

Participants with a normal BMI consumed more portions of fruits (*p* = 0.034), vegetables (*p* = 0.003), and legumes/pulses (*p* < 0.001) per week during lockdown compared to the period after lockdown (Table 4). Similarly, overweight participants reported consuming fewer portions of legumes/pulses per week after lockdown compared to the period during lockdown (*p* = 0.007). About 7% (*n* = 54) of the individuals with normal weight never consumed potatoes during lockdown, while after lockdown the corresponding percentage decreased (*n* = 33, 5.7%) (*p* < 0.001). In contrast, the percentage of overweight (4.4% and 7.3% during and post-lockdown, respectively, *p* < 0.001) and obese people (3.1% and 9.4% during and post-lockdown, respectively, *p* = 0.002) who never consumed potatoes during lockdown was smaller than the percentage after lockdown.

Statistically significant differences between the consumption of fish during and after lockdown were identified among individuals with normal BMI (*p* = 0.005) and overweight participants (*p* = 0.017). In both categories, the consumption of fish was lower during lockdown than after lockdown. Furthermore, we only found a statistically significant association (*p* = 0.014) between the consumption of red meat and meat products during and after lockdown in overweight individuals, with the consumption after lockdown being higher than during lockdown. Moreover, we found statistically significant differences between the consumption of poultry during and after lockdown among normal BMI (*p* = 0.035), overweight (*p* < 0.001), and obese (*p* = 0.010) participants. In addition, a higher consumption of full-fat dairy products was identified after lockdown than during lockdown in overweight participants (*p* < 0.001). We found more normal BMI (*p* < 0.001), overweight (*p* = 0.014), and obese (*p* = 0.001) individuals who consumed olive oil daily during lockdown compared to the period after lockdown. Among obese individuals, we also found that 91.0% (*n* = 201) consumed less than 300 mL of alcohol during lockdown, while the corresponding percentage after lockdown was 81.4% (*n* = 101) (*p* = 0.029). Moreover, the number of obese individuals who consumed 300–500 mL of alcohol after lockdown was more than that during lockdown. Statistically significant differences between the frequency of delivery during and after lockdown were identified among all BMI categories (*p* < 0.001) (Table 4).

### 3.5. Physical Activity and Dietary Habits

We reported statistically significant associations for the consumption of non-refined cereals (*p* < 0.001), fruits (*p* < 0.001), vegetables (*p* < 0.001), and potatoes (*p* = 0.005) among physical activity categories during the lockdown (Supplementary Table S3). Physically active participants consumed fewer portions of non-refined cereals and potatoes and more portions of fruits and vegetables compared to not-adequately physically active participants during the lockdown. During the post-lockdown period, we found that physically active participants consumed more portions of non-refined cereals (*p* < 0.001), fruits (*p* < 0.001), vegetables (*p* = 0.017), legumes/pulses (*p* = 0.001), fish (*p* < 0.001), and olive oil (*p* = 0.032), and fewer portions of potatoes (*p* < 0.001), poultry (*p* = 0.048), full-fat dairy products (*p* = 0.001), and alcohol (*p* = 0.004) compared to not-adequately physically active participants.

## 4. Discussion

To the best to our knowledge, this was the first study that investigated the differences in dietary habits and lifestyle characteristics during and after the lockdown due to the COVID-19 pandemic in Cyprus. In particular, the objective of this study was to identify the changes in specific food consumption, alcohol intake, use of delivery services, coffee and food supplement consumption, smoking habits, physical activity level, and BMI in total samples as well as separately between males and females and among BMI categories. We found that people consumed more fruits, vegetables, legumes/pulses, fish, and poultry during lockdown than they did thereafter. In addition, we found a greater daily intake of olive oil and a lower consumption of alcohol during the lockdown period compared to the post-lockdown period. During lockdown, most participants never or rarely used delivery services, whist many of the participants after lockdown used delivery services 1–3 times per month. Moreover, during lockdown, more participants were physically active, compared to the participants after lockdown. Furthermore, when compared to those with a normal BMI, more overweight and obese respondents ordered food 1–2 times per week in both periods.

According to our findings, the consumption of fruits, vegetables, and legumes was higher during the lockdown compared to the post-lockdown period. Our results are comparable with other studies, which found an increase in fruit, vegetable, and legume consumption during the lockdown [19,35,36,37,38]. Our findings are also consistent with the literature, which supports that people consumed more healthy foods such as fruits and vegetables during lockdown [39,40,41,42]. In contrast, an Italian study investigated the dietary habits during the COVID-19 lockdown and reported that there was a lack of food access, which led to lower consumption of fresh fruits and higher concentrations of processed foods [20]. Moreover, another study conducted in Lebanon during lockdown reported a low consumption of fruits and vegetables during the COVID-19 pandemic [3]. A study in Canada reported that dietary habits changed during the COVID-19 pandemic, as the consumption of snacks and home-cooked meals increased [43,44]. Moreover, a recent study discovered that increased meal preparation time is linked to a higher intake of fruits, vegetables, and legumes [19]. Therefore, this could explain the lower consumption of those foods after the lockdown period when Cypriots physically returned to work and the preparation time for home-cooked meals was limited.

Apart from the increased percentage of home-cooked meals during the lockdown period, participants might have had healthier dietary habits due to the lower use of delivery services. Almost 45% of participants in this study did not order food during lockdown period although delivery services were available, while during the post-lockdown period the corresponding percentage decreased to 18%. This is probably because during the lockdown period an individual had increased available time for preparing meals. Our finding is in agreement with other research studies in Spain [19] and Kuwait [45] which found that 82% and about 69% did not order delivery food at home during the pandemic, respectively. Moreover, a recent observational study that investigated the dietary habits before and during the COVID-19 period in several European countries found that the change in eating out once a week in Austria, the United Kingdom, and Poland has decreased [16]. Specifically, the differences in eating out before and during the pandemic were 46.74%, 40.84% and 9.58% among Austrian, British, and Polish respondents, respectively [16]. In Cyprus, restaurants and cafes were closed during the lockdown period and only some restaurants provided home delivery of food. Thus, the reduced use of delivery services may be due to the limited working hours of restaurants or to the fear of transmission of the COVID-19 virus via food packaging.

We reported participants eating fish and poultry more often during the lockdown period compared to the period after the restrictive measures. Our finding is consistent with other research studies, which reported that the consumption of fish increased during lockdown [37,46,47]. In contrast, other studies among Chinese [48] and Poles [49] reported a low consumption of fish during the lockdown period, mainly due to the limited availability of fresh fish. In Spain, a Mediterranean country, a greater intake of fish and fishery products was reported [19]. We did not report any change in the consumption of meat and meat products during and after lockdown. However, in Spain [19] and among the Kurdish population [50] during lockdown, a larger consumption of red meat was found, while a reduced consumption of red meat was identified in Croatia [18], Denmark [46], and Italy [47]. However, we found differences in meat consumption among females and males during as well as after lockdown. More males consumed more than four portions of meat and meat products in both periods, with the largest difference being found after lockdown.

Furthermore, a greater proportion of respondents reported consuming olive oil every day during the lockdown than after the restricted measures were lifted. We also reported more females who consumed olive oil daily during and after lockdown as well. In the literature it is supported that females have healthier dietary habits compared to males in general [8,17,51,52,53], and this could explain the fact that females consumed olive oil daily during and after lockdown. Moreover, our results are similar to another study in Spain which investigated the change in dietary habits due to COVID-19 after the lockdown period, and found that 98.2% of the respondents consumed olive oil daily [37]. Similarly, other studies reported an increase in olive oil consumption and use during the lockdown period [37,46,53]. On the other hand, in Kuwait, it was found that the most common fat for cooking was vegetable oil, followed by olive oil, before and during lockdown [45]. As mentioned above, during lockdown people preferred home-cooked meals, therefore the higher consumption of olive oil in the Cypriot population during lockdown compared to after lockdown could be explained by the fact that olive oil is the main source of dietary fat in the Cypriot diet, which is usually accompanied by the consumption of large portions of vegetables, either in the form of cooked foods or salads.

During the lockdown, fewer people reported taking vitamin D, compared to the period post-lockdown. It has been reported that vitamin D reduces the risk of acute respiratory tract infections from COVID-19 and represses the risk of inflammatory cytokine production [54]. Studies investigated the role of vitamin D in the prevention of COVID-19 and the results showed that sunlight as well vitamin D might decrease the risk of COVID-19 cases and deaths [51]. During the lockdown period, Cypriots were not exposed to sunlight for many hours per day even though it was summer. However, after lockdown, although it was autumn, people were exposed in sunlight for several hours, since in Cyprus it is sunny almost every day, even during winter.

Our findings showed that more than 90% of the respondents consumed less than 300 mL of alcohol per day during lockdown, while consumption in the post-lockdown period increased. Our results are comparable to those of Spanish [52,53] and Italian population [20] studies which found a large decrease in alcohol consumption during the lockdown period. In Spain and Italy, alcohol drinking is customary during traditional meals with family and friends, which were not available during the lockdown period due to restrictions on social gatherings [55]. Similarly, the culture of Cyprus consists of food gatherings with friends and family. In contrast, other research studies found an increase in alcohol consumption during lockdown [37,46,56].

The current study found a larger percentage of overweight and obese individuals during lockdown than after lockdown. We also found that overweight people consumed more legumes/pulses, potatoes, and fish, while there was less consumption of meat, meat products, and chicken during lockdown than after. Our findings indicate that overweight people had healthier dietary habits during the lockdown period. Hence, this decrease might be related to the healthier dietary and lifestyle habits adapted by Cypriots during lockdown. Moreover, it has been shown that obesity is a dominant predictor of increased mortality due to COVID-19 [57], so it was important that individuals with a high BMI adopted healthier food choices during the pandemic. According to our findings, more overweight and obese respondents ordered food 1–2 times per week than those with a normal BMI. This finding agrees with a study that investigated the dietary choices and habits during the COVID-19 lockdown in Poland [49]. Moreover, the finding that more overweight and obese respondents ordered food compared to those with a normal BMI could be attributed to the fact that individuals with a higher BMI are more likely to eat in order to control stress [58].

We found a larger percentage of physically active participants during lockdown than in the post-lockdown period. Our findings vary from those of other studies that found a decrease in physical activity during lockdown measures due to COVID-19 [39,49,59]. In particular, these findings were reported in Saudi Arabia [35], Kuwait [45], and Spain [60], so they could be attributed to a lack of access to outdoor physical activity or a lack of space at home. However, in Cyprus, most of the year’s pleasant and sunny weather, as well as the available outdoor space at the majority of homes, allowed for increased physical activity during lockdown. Moreover, gyms and sports centers promoted online training programs through social media platforms, so the population of Cyprus was able to access those training programs at home. In fact, a cross-sectional study which investigated the lifestyle habits of adults during the COVID-19 pandemic lockdown in Cyprus found that physical activity habits did not change during lockdown, however there was an increase in walking’s energy expenditure [5]. It has been reported that lower physical activity levels combined with increased levels of stress could cause higher alcohol consumption and smoking [61,62,63].

Our findings revealed a larger percentage of current smokers after lockdown than during lockdown. This finding is in line with earlier research that examined lifestyle patterns among the Spanish population following lockdown and found a similar percentage of non-smokers before and during lockdown [19]. One possible explanation is that smoking increases the occurrence of several respiratory diseases [64], therefore people may quit smoking at that time. On the other hand, two online surveys in Israel [65] and Italy [59] found that current smokers reported an increase in their smoking frequency. The last one observed that the increase in cigarette consumption among current smokers during lockdown might be due to an increase in mental distress [59].

This study has some limitations. Firstly, only associations between the groups of interest were estimated due to the cross-sectional design of the study. Secondly, as a convenience sample size was recruited, we cannot exclude the possibility that individuals without access to technology are probably underrepresented in our sample. Thirdly, we could not estimate how many individuals might have seen the link to the survey and declined to participate, therefore the response rate was not able to be calculated. Fourthly, we did not include questions about the participants’ health and the use of specific diets. Finally, the study participants’ level of physical activity was not assessed using a validated questionnaire.

## 5. Conclusions

To the best to our knowledge, this was the first study to investigate the differences in dietary habits and lifestyle characteristics during and after lockdown due to the COVID-19 pandemic in Cyprus. The results in the present study suggested a higher consumption of fruits, vegetables, legumes/pulses, fish and poultry, increased home-cooked meals, a greater daily intake of olive oil, a lower consumption of alcohol, and a limited use of delivery services during the lockdown period compared to the post-lockdown period. In addition, an important proportion of the population had healthier lifestyle habits, including being involved in physical activity, during the COVID-19 pandemic, than after. It is critical to encourage the population of Cyprus to maintain the positive dietary and lifestyle habits they developed during the lockdown in their daily lives after the lockdown, which could have long-term health benefits, such as lowering the risk of diet-related diseases such as obesity.

## Figures and Tables

**Table 1:** Sociodemographic characteristics of the first cross-sectional survey (during lockdown) and the second cross-sectional survey (post-lockdown).

Characteristics	During Lockdown (*N* = 1460, 58.3%)	Post-Lockdown (*N* = 1043, 41.7%)	*p*-Value
**Mean Age** (SD)	35.3 ± 11.4	32.2 ± 11.1	**<0.001 ^g^**
**Gender** [*N* ^a^ (%)]			
Female	875 (60.5)	719 (68.9)	**<0.001 ^h^**
Male	571 (39.5)	324 (31.1)	
**Geographical area** [*N* ^b^ (%)]			
Nicosia	779 (53.7)	593 (56.9)	**0.013 ^h^**
Limassol	337 (23.2)	224 (21.5)	
Larnaca	201 (13.9)	120 (11.5)	
Paphos	89 (6.1)	87 (8.3)	
Ammochostos	45 (6.1)	19 (1.8)	
**Residency** [*N* ^c^ (%)]			
Urban	1208 (83.3)	836 (80.2)	**0.043 ^h^**
Rural	242 (16.7)	207 (19.8)	
**Marital status** [*N* ^d^ (%)]			
Married/In cohabitation	746 (51.6)	455 (43.9)	**<0.001 ^h^**
Unmarried	618 (42.7)	538 (51.9)	
Divorced/separated/widowed	82 (5.7)	43 (4.2)	
**Having children** [*N* ^a^ (%)]			
No	765 (52.7)	649 (62.6)	**<0.001 ^h^**
Yes	687 (47.3)	388 (37.4)	
**Education** [*N* ^e^ (%)]			
Primary education	2 (0.1)	0 (0.0)	**<0.001 ^h^**
Secondary education	251 (17.2)	310 (29.7)	
Higher education	1205 (82.7)	733 (70.3)	
**Annual income** [*N* ^f^ (%)]			
Low (≤€6500)	309 (21.2)	230 (22.2)	**<0.001 ^h^**
Moderate (€6500–19,500)	502 (34.5)	551 (53.2)	
High (>€19,500)	645 (44.3)	255 (24.6)	

Abbreviations: SD = standard deviation; ^a^ *N* = 2489; ^b^ *N* = 2494; ^c^ *N* = 2493; ^d^ *N* = 2482; ^e^ *N* = 2501; ^f^ *N* = 2492; ^g^ Differences between dietary habits among the first cross-sectional study and the second cross-sectional study were tested using t-test; ^h^ Differences between dietary habits among the first cross-sectional study and the second cross-sectional study were tested using chi^2^ test. Bold values indicate statistically significant associations (*p* < 0.05).

**Table 2:** Dietary habits in the first cross-sectional survey (during lockdown) and the second cross-sectional survey (post-lockdown) and for females and males separately.

	Cross-Sectional Study			During Lockdown (*N* = 1460)			Post-Lockdown (*N* = 1043)		
Dietary Habits	During Lockdown (*N* = 1460, 58.3%)	Post-Lockdown (*N* = 1043, 41.7%)	*p*-Value ^j^	Female (*N* = 875, 59.9%)	Male (*N* = 57, 40.1%)	*p*-Value ^k^	Female (*N* = 719, 68.9%)	Male (*N* = 324, 31.1%)	*p*-Value ^k^
**Non-refined cereals** [*N* ^a^ (%)]									
Never	251 (17.2)	196 (18.9)	0.214	149 (59.8)	100 (40.2)	0.777	117 (56.7)	79 (40.3)	**0.026**
1–6 portions/week	735 (50.3)	522 (50.2)		444 (60.9)	285 (39.1)		379 (72.6)	143 (27.4)	
7–12 portions/week	293 (20.1)	177 (17.0)		176 (60.7)	114 (39.3)		125 (70.6)	52 (29.4)	
13–18 portions/week	111 (7.6)	85 (8.2)		63 (57.8)	46 (42.2)		55 (64.7)	30 (35.3)	
19–31 portions/week	55 (3.8)	41 (3.9)		36 (66.7)	18 (33.3)		30 (73.2)	11 (26.8)	
>32 portions/week	15 (1.0)	19 (1.8)		7 (46.7)	8 (53.3)		12 (63.2)	7 (36.8)	
**Fruits** [*N* ^b^ (%)]									
Never	62 (4.3)	67 (6.4)	**0.042**	41 (67.2)	20 (32.8)	0.115	45 (67.2)	22 (32.8)	0.355
1–4 portions/week	466 (32.0)	366 (35.1)		288 (62.6)	172 (37.4)		246 (67.2)	120 (32.8)	
5–8 portions/week	395 (27.1)	271 (26.0)		223 (57.0)	168 (43.0)		194 (71.6)	77 (28.4)	
9–15 portions/week	309 (21.2)	185 (17.8)		178 (58.0)	129 (42.0)		135 (73.0)	50 (27.0)	
16–21 portions/week	129 (8.8)	87 (8.4)		88 (68.8)	40 (31.2)		53 (60.9)	34 (39.1)	
>22 portions/week	96 (6.6)	66 (6.3)		55 (57.3)	41 (42.7)		45 (68.2)	21 (31.8)	
**Vegetables** [*N* ^c^ (%)]									
Never	39 (2.7)	30 (2.9)	**<0.001**	24 (61.5)	15 (38.5)	0.345	19 (63.3)	11 (36.7)	0.189
1–6 portions/week	509 (34.9)	383 (36.7)		293 (58.5)	208 (41.5)		270 (70.5)	113 (29.5)	
7–12 portions/week	465 (31.9)	286 (27.4)		280 (60.9)	180 (39.1)		196 (68.5)	90 (31.5)	
13–20 portions/week	269 (18.5)	157 (15.1)		156 (58.2)	112 (41.8)		96 (61.2)	61 (38.8)	
21–32 portions/week	120 (8.2)	116 (11.1)		82 (68.3)	38 (31.7)		85 (73.3)	31 (26.7)	
>33 portions/week	55 (3.8)	71 (6.8)		37 (67.3)	18 (32.7)		53 (74.7)	18 (25.3)	
**Legumes/pulses** [*N* ^d^ (%)]									
Never	76 (5.1)	101 (9.6)	**<0.001**	45 (59.2)	31 (40.8)	0.074	75 (74.3)	26 (25.7)	0.054
Less than 1 portion/week	217 (14.9)	222 (21.4)		131 (62.1)	80 (37.9)		159 (71.6)	63 (28.4)	
1–2 portions/week	730 (50.0)	510 (49.1)		451 (62.2)	274 (37.8)		354 (69.4)	156 (30.6)	
3–4 portions/week	378 (25.9)	170 (16.4)		217 (57.7)	159 (42.3)		112 (65.9)	58 (34.1)	
5–6 portions/week	45 (3.1)	28 (2.7)		28 (62.2)	17 (37.8)		13 (46.4)	15 (53.6)	
>6 portions/week	14 (1.0)	8 (0.8)		3 (23.1)	10 (76.9)		4 (50.0)	4 (50.0)	
**Potatoes** [*N* ^e^ (%)]									
Never	84 (5.8)	67 (6.5)	**<0.001**	57 (69.5)	25 (30.5)	**0.020**	52 (77.6)	15 (22.4)	**<0.001**
1–4 portions/week	1099 (75.3)	661 (63.6)		653 (60.0)	435 (40.0)		489 (74.0)	172 (26.0)	
5–8 portions/week	184 (12.6)	159 (15.3)		115 (62.8)	68 (37.2)		97 (61.0)	62 (39.0)	
9–12 portions/week	62 (4.3)	78 (7.5)		29 (46.8)	33 (53.2)		46 (59.0)	32 (41.0)	
13–18 portions/week	22 (1.5)	51 (4.9)		13 (59.1)	9 (40.9)		26 (51.0)	25 (49.0)	
>18 portions/week	8 (0.5)	23 (2.2)		8 (100.0)	0 (0.0)		7 (30.4)	16 (70.6)	
**Fish** [*N* ^f^ (%)]									
Never	140 (9.6)	149 (14.3)	**<0.001**	82 (59.8)	55 (40.2)	0.485	95 (63.8)	54 (36.2)	0.506
Less than 1 portion/week	465 (31.9)	399 (38.3)		277 (60.2)	183 (39.8)		281 (70.4)	118 (29.6)	
1–2 portions/week	670 (45.9)	365 (35.0)		401 (60.4)	263 (39.6)		259 (71.0)	106 (29.0)	
3–4 portions/week	153 (10.5)	104 (10.0)		94 (61.4)	59 (38.6)		69 (66.3)	35 (33.7)	
5–6 portions/week	25 (1.7)	22 (2.1)		19 (76.0)	6 (24.0)		13 (59.1)	9 (40.9)	
>6 portions/week	6 (0.4)	3 (0.3)		2 (33.3)	4 (66.7)		2 (66.7)	1 (33.3)	
**Meat and meat products** [*N* ^g^ (%)]									
1 or less than 1 portion/week	671 (46.1)	477 (46.1)	0.634	471 (70.5)	197 (29.5)	**<0.001**	382 (80.1)	95 (19.9)	**<0.001**
2–3 portions/week	470 (32.3)	312 (30.2)		248 (53.5)	216 (46.5)		212 (68.0)	100 (32.0)	
4–5 portions/week	187 (12.9)	137 (13.3)		99 (53.5)	86 (46.5)		74 (54.0)	63 (46.0)	
6–7 portions/week	72 (5.0)	61 (5.9)		36 (51.4)	34 (48.6)		24 (39.3)	37 (60.7)	
8–10 portions/week	41 (2.8)	33 (3.2)		18 (45.0)	22 (55.0)		12 (36.4)	21 (63.3)	
>10 portions/week	13 (0.9)	14 (1.3)		1 (7.7)	12 (92.3)		7 (50.0)	7 (50.0)	
**Poultry** [*N* ^h^ (%)]									
3 or less than 3 portions/week	812 (55.9)	469 (45.5)	**<0.001**	522 (64.8)	284 (35.2)	**<0.001**	348 (74.2)	121 (25.8)	**<0.001**
4–5 portions/week	352 (24.2)	241 (23.3)		206 (58.9)	144 (41.1)		176 (73.0)	65 (27.0)	
5–6 portions/week	134 (9.3)	120 (11.6)		65 (49.6)	66 (50.4)		79 (65.8)	41 (34.2)	
7–8 portions/week	93 (6.4)	99 (9.6)		50 (55.0)	41 (45.0)		55 (55.6)	44 (44.4)	
9–10 portions/week	41 (2.8)	69 (6.7)		24 (60.0)	16 (40.0)		36 (52.2)	33 (47.8)	
>10 portions/week	20 (1.4)	34 (3.3)		4 (20.0)	16 (80.0)		14 (41.2)	20 (58.8)	
**Full-fat dairy products** [*N* ^i^ (%)]									
10 or less than 10 portions/week	994 (68.3)	669 (64.5)	**0.004**	600 (61.0)	384 (39.0)	0.417	499 (74.6)	170 (25.4)	**<0.001**
11–15 portions/week	252 (17.3)	175 (16.9)		156 (62.4)	94 (37.6)		113 (64.6)	62 (35.4)	
16–20 portions/week	108 (7.4)	76 (7.3)		59 (55.1)	48 (44.9)		44 (57.9)	32 (42.1)	
21–28 portions/week	54 (3.7)	54 (5.2)		33 (62.3)	20 (37.7)		30 (55.6)	24 (44.4)	
29–30 portions/week	24 (1.7)	40 (3.9)		16 (66.7)	8 (33.3)		18 (45.0)	22 (55.0)	
>30 portions/week	23 (1.6)	23 (2.2)		10 (43.5)	13 (56.5)		11 (47.8)	12 (52.2)	

During and after lockdown respectively: ^a^ *N* = 1460 and 1040; ^b^ *N* = 1457 and 1042; ^c^ *N* = 1457 and 1043; ^d^ *N* = 1460 and 1039; ^e^ *N* = 1459 and 1039; ^f^ *N* = 1459 and 1042; ^g^ *N* = 1454 and 1034; ^h^ *N* = 1452 and 1032; ^i^ *N* = 1455 and 1037; ^j^ Differences between dietary habits among the first cross-sectional study and the second cross-sectional study were tested using chi2 test; ^k^ Differences between dietary habits of females and males were tested using chi2 test. Bold values indicate statistically significant associations (*p* < 0.05).

**Table 3:** Lifestyle habits in the first cross-sectional survey (during lockdown) and the second cross-sectional survey (post-lockdown).

Lifestyle Habits	During Lockdown (*N* = 1460, 58.3%)	After Lockdown (*N* = 1043, 41.7%)	*p*-Value ^e^
**Smoking habits** [*N* ^a^ (%)]			
Non-smoker	949 (65.1)	686 (66.0)	**<0.001**
Current smoker	323 (22.2)	279 (26.8)	
Past smoker	185 (12.7)	75 (7.2)	
**Physical activity** [*N* ^b^ (%)]			
Not adequately physical active	496 (34.1)	463 (44.5)	**<0.001**
Physical active	959 (65.9)	578 (55.5)	
**Hours of exercising** [*N* ^c^ (%)]			
Less than 1 h	294 (24.8)	172 (24.2)	**0.039**
1–3 h	371 (31.3)	270 (38.0)	
3–6 h	340 (28.7)	181 (25.5)	
6–9 h	123 (10.4)	60 (8.4)	
More than 9 h	57 (4.8)	28 (3.9)	
**BMI** [*N* ^d^ (%)]			
Underweight	71 (4.9)	64 (6.2)	**0.010**
Normal weight	736 (50.9)	584 (56.4)	
Overweight	411 (28.4)	261 (25.2)	
Obese	228 (15.8)	127 (12.2)	

During and after lockdown respectively: ^a^ *N* = 1457 and 1040; ^b^ *N* = 1455 and 1041; ^c^ *N* = 1185 and 711; ^d^ *N* = 1446 and 1036; ^e^ Differences between dietary habits among first cross-sectional study and second cross-sectional study were tested using chi2 test. Bold values indicate statistically significant associations (*p* < 0.05).

**Table 4:** Dietary habits in the first cross-sectional survey (during lockdown) and the second cross-sectional survey (post-lockdown) among BMI categories.

	Underweight (*N* = 135, 5.4%)			Normal Weight (*N* = 1320, 53.2%)			Overweight (*N* = 672, 27.1%)			Obese (*N* = 355, 14.3%)		
	During Lockdown (*N* = 71, 52.6%)	Post-Lockdown (*N* = 64, 47.4%)	*p*-Value ^q^	During Lockdown (*N* = 736, 55.7%)	Post-Lockdown (*N* = 584, 44.3%)	*p*-Value ^q^	During Lockdown (*N* = 411, 61.2%)	Post-Lockdown (*N* = 261, 38.8%)	*p*-Value ^q^	During Lockdown (*N* = 228, 64.2%)	Post-Lockdown (*N* = 127, 35.8%)	*p*-Value ^q^
**Non-refined cereals** [*N* ^a^ (%)]												
Never	15 (21.1)	12 (19.0)	0.849	122 (16.5)	112 (19.2)	0.041	76 (18.5)	43 (16.5)	0.871	35 (15.3)	28 (22.0)	0.172
1–6 portions/week	34 (47.9)	37 (58.7)		368 (50.0)	290 (49.7)		204 (49.6)	131 (50.4)		122 (53.5)	59 (46.5)	
7–12 portions/week	10 (14.1)	7 (11.1)		158 (21.5)	89 (15.3)		84 (20.4)	57 (21.9)		38 (16.7)	24 (18.9)	
13–18 portions/week	7 (9.9)	4 (6.4)		52 (7.1)	56 (9.6)		27 (6.6)	15 (5.8)		24 (10.5)	9 (7.1)	
19–31 portions/week	4 (5.6)	2 (3.2)		26 (3.5)	25 (4.3)		16 (3.9)	9 (3.5)		9 (4.0)	5 (3.9)	
>32 portions/week	1 (1.4)	1 (1.6)		10 (1.4)	11 (1.9)		4 (1.0)	5 (1.9)		0 (0.0)	2 (1.6)	
**Fruits** [*N* ^b^ (%)]												
Never	6 (8.6)	4 (6.3)	0.770	32 (4.3)	46 (7.9)	**0.034**	16 (3.9)	12 (4.6)	0.167	8 (3.5)	5 (3.9)	0.996
1–4 portions/week	26 (37.1)	21 (32.8)		226 (30.7)	203 (34.8)		131 (31.9)	94 (36.0)		79 (34.8)	46 (36.2)	
5–8 portions/week	17 (24.3)	23 (35.9)		201 (27.4)	135 (23.2)		111 (27.1)	75 (28.7)		58 (25.5)	33 (26.0)	
9–15 portions/week	13 (18.6)	11 (17.2)		153 (20.8)	113 (19.4)		93 (22.6)	37 (14.2)		49 (21.6)	24 (18.9)	
16–21 portions/week	4 (5.7)	2 (3.1)		71 (9.7)	52 (8.9)		37 (9.0)	24 (9.2)		16 (7.1)	9 (7.1)	
>22 portions/week	4 (5.7)	3 (4.7)		52 (7.1)	34 (5.8)		23 (5.5)	19 (7.3)		17 (7.5)	10 (7.9)	
**Vegetables** [*N* ^c^ (%)]												
Never	3 (4.2)	4 (6.3)	0.655	22 (3.0)	21 (3.6)	**0.003**	7 (1.7)	3 (1.1)	0.143	5 (2.2)	2 (1.6)	0.096
1–6 portions/week	27 (38.0)	30 (46.9)		244 (33.2)	220 (37.7)		147 (35.9)	83 (31.8)		86 (37.7)	49 (38.6)	
7–12 portions/week	20 (28.2)	11 (17.2)		233 (31.7)	159 (27.2)		133 (32.4)	77 (29.5)		76 (33.3)	35 (27.6)	
13–20 portions/week	11 (15.5)	12 (18.7)		143 (19.5)	79 (13.5)		73 (17.8)	47 (18.0)		38 (16.7)	17 (13.4)	
21–32 portions/week	8 (11.3)	5 (7.8)		61 (8.3)	69 (11.8)		35 (8.5)	31 (11.9)		16 (7.0)	11 (8.7)	
>33 portions/week	2 (2.8)	2 (3.1)		31 (4.3)	36 (6.2)		15 (3.7)	20 (7.7)		7 (3.1)	13 (10.1)	
**Legumes/pulses** [*N* ^d^ (%)]												
Never	4 (5.6)	3 (4.7)	0.815	42 (5.7)	58 (10.0)	**<0.001**	14 (3.4)	21 (8.1)	**0.007**	14 (6.1)	19 (15.2)	0.061
Less than 1 portion/week	11 (15.5)	15 (23.4)		103 (14.0)	131 (22.4)		57 (13.9)	45 (17.3)		44 (19.3)	29 (23.2)	
1–2 portions/week	38 (53.5)	33 (51.6)		359 (48.8)	277 (47.4)		217 (52.8)	143 (55.0)		111 (48.7)	55 (44.0)	
3–4 portions/week	15 (21.1)	11 (17.2)		198 (26.9)	96 (16.4)		112 (27.2)	43 (16.5)		48 (21.0)	18 (14.4)	
5–6 portions/week	3 (4.3)	2 (3.1)		26 (3.5)	18 (3.1)		7 (1.7)	5 (1.9)		9 (4.0)	3 (2.4)	
>6 portions/week	0 (0.0)	0 (0.0)		8 (1.1)	4 (0.7)		4 (1.0)	3 (1.2)		2 (0.9)	1 (0.8)	
**Potatoes** [*N* ^e^ (%)]												
Never	5 (7.1)	3 (4.8)	0.913	54 (7.3)	33 (5.7)	**<0.001**	18 (4.4)	19 (7.3)	**<0.001**	7 (3.1)	12 (9.4)	**0.002**
1–4 portions/week	46 (64.8)	38 (61.3)		548 (74.5)	385 (66.0)		324 (78.8)	161 (61.9)		170 (74.9)	73 (57.5)	
5–8 portions/week	13 (18.3)	14 (22.6)		97 (13.2)	90 (15.4)		42 (10.2)	35 (13.5)		30 (13.2)	19 (15.0)	
9–12 portions/week	3 (4.2)	4 (6.5)		24 (3.3)	47 (8.1)		23 (5.6)	18 (6.9)		11 (4.8)	8 (6.3)	
13–18 portions/week	4 (5.6)	3 (4.8)		9 (1.2)	22 (3.8)		2 (0.5)	17 (6.5)		7 (3.1)	8 (6.3)	
>18 portions/week	0 (0.0)	0 (0.0)		4 (0.5)	6 (1.0)		2 (0.5)	10 (3.9)		2 (0.9)	7 (5.5)	
**Fish** [*N* ^f^ (%)]												
Never	7 (9.9)	9 (14.1)	0.107	72 (9.8)	78 (13.4)	**0.005**	34 (8.3)	38 (14.6)	**0.017**	27 (11.9)	20 (15.8)	0.138
Less than 1 portion/week	17 (23.9)	25 (39.1)		232 (31.5)	226 (38.7)		125 (30.4)	94 (36.2)		84 (37.0)	53 (41.7)	
1–2 portions/week	38 (53.5)	20 (31.1)		333 (45.2)	211 (36.1)		199 (48.4)	96 (36.9)		93 (41.0)	36 (28.3)	
3–4 portions/week	7 (9.9)	9 (14.1)		82 (11.1)	53 (9.1)		47 (11.4)	26 (10.0)		17 (7.5)	16 (12.6)	
5–6 portions/week	2 (2.8)	1 (1.6)		15 (2.1)	14 (2.4)		4 (1.0)	5 (1.9)		4 (1.7)	2 (1.6)	
>6 portions/week	0 (0.0)	0 (0.0)		2 (0.3)	2 (0.3)		2 (0.5)	1 (0.4)		2 (0.9)	0 (0.0)	
**Meat and meat products** [*N* ^g^ (%)]												
1 or less than 1 portion/week	36 (50.7)	34 (54.8)	0.097	362 (49.2)	287 (49.6)	0.862	177 (43.4)	99 (37.9)	**0.014**	89 (39.3)	55 (44.0)	0.074
2–3 portions/week	19 (26.8)	20 (32.3)		223 (30.4)	181 (31.3)		145 (35.5)	82 (31.4)		80 (35.2)	27 (21.6)	
4–5 portions/week	11 (15.5)	2 (3.2)		88 (12.0)	68 (11.7)		56 (13.7)	40 (15.3)		28 (12.3)	24 (19.2)	
6–7 portions/week	5 (7.0)	4 (6.5)		32 (4.4)	27 (4.7)		15 (3.7)	21 (8.1)		20 (8.8)	9 (7.2)	
8–10 portions/week	0 (0.0)	2 (3.2)		21 (2.9)	13 (2.3)		14 (3.4)	14 (5.4)		6 (2.6)	6 (4.8)	
>10 portions/week	0 (0.0)	0 (0.0)		8 (1.1)	3 (0.5)		1 (0.3)	5 (1.9)		4 (1.8)	4 (3.2)	
**Poultry** [*N* ^h^ (%)]												
3 or less than 3 portions/week	40 (56.4)	26 (41.9)	0.415	411 (56.2)	289 (50.0)	**0.035**	235 (57.3)	98 (37.7)	**<0.001**	119 (52.7)	53 (42.4)	**0.010**
4–5 portions/week	14 (19.7)	17 (27.5)		173 (23.7)	138 (23.9)		107 (26.1)	51 (19.6)		53 (23.4)	33 (26.4)	
5–6 portions/week	9 (12.7)	9 (14.5)		77 (10.5)	62 (10.7)		30 (7.3)	33 (12.7)		16 (7.1)	16 (12.8)	
7–8 portions/week	4 (5.6)	8 (12.9)		42 (5.7)	52 (9.0)		22 (5.4)	32 (12.3)		25 (11.1)	6 (4.8)	
9–10 portions/week	3 (4.2)	1 (1.6)		18 (2.5)	27 (4.7)		12 (2.9)	27 (10.4)		8 (3.5)	13 (10.4)	
>10 portions/week	1 (1.4)	1 (1.6)		10 (1.4)	10 (1.7)		4 (1.0)	19 (7.3)		5 (2.2)	4 (3.2)	
**Full-fat dairy products** [*N* ^i^ (%)]												
10 or less than 10 portions/week	36 (50.7)	39 (62.9)	0.639	495 (67.5)	393 (67.5)	0.865	306 (74.6)	154 (59.5)	**<0.001**	146 (64.3)	79 (62.2)	0.066
11–15 portions/week	17 (23.9)	11 (17.7)		135 (18.4)	99 (17.0)		58 (14.1)	46 (17.8)		40 (17.6)	18 (14.2)	
16–20 portions/week	11 (15.5)	8 (12.9)		50 (6.8)	41 (7.0)		27 (6.6)	18 (6.9)		19 (8.4)	8 (6.3)	
21–28 portions/week	5 (7.1)	4 (6.5)		27 (3.7)	28 (4.8)		11 (2.7)	18 (6.9)		11 (4.9)	4 (3.1)	
29–30 portions/week	1 (1.4)	0 (0.0)		16 (2.2)	15 (2.6)		2 (0.5)	15 (5.8)		5 (2.2)	9 (7.1)	
>30 portions/week	1 (1.4)	0 (0.0)		10 (1.4)	6 (1.1)		6 (1.5)	8 (3.1)		6 (2.6)	9 (7.1)	
**Olive oil** [*N* ^j^ (%)]												
Never	1 (1.4)	1 (1.6)	0.160	14 (1.9)	5 (0.9)	**<0.001**	1 (0.2)	4 (1.5)	**0.014**	2 (0.9)	4 (3.1)	**0.001**
Rarely	10 (14.1)	5 (7.8)		47 (6.4)	69 (11.9)		30 (7.3)	28 (10.8)		8 (3.5)	17 (13.4)	
Less than 1 portion/week	2 (2.9)	8 (12.5)		34 (4.6)	37 (6.4)		19 (4.6)	16 (6.2)		16 (7.0)	4 (3.1)	
1–3 portions/week	14 (19.7)	19 (29.7)		163 (22.2)	169 (29.1)		91 (22.3)	74 (28.6)		54 (23.7)	35 (27.6)	
3–5 portions/week	17 (23.9)	12 (18.7)		152 (20.6)	119 (20.5)		101 (24.6)	58 (22.4)		58 (25.4)	35 (27.6)	
Daily	27 (38.0)	19 (29.7)		326 (44.3)	182 (31.2)		168 (41.0)	79 (30.5)		90 (39.5)	32 (25.2)	
**Alcohol** [*N* ^k^ (%)]												
<300 mL	67 (94.4)	55 (88.7)	0.361	671 (92.0)	512 (88.9)	0.281	357 (88.6)	220 (85.3)	0.609	201 (91.0)	101 (81.4)	**0.029**
300 mL	2 (2.8)	5 (8.1)		32 (4.4)	36 (6.2)		25 (6.2)	21 (8.1)		13 (5.9)	10 (8.1)	
400 mL	1 (1.4)	2 (3.2)		10 (1.4)	12 (2.1)		10 (2.5)	8 (3.1)		6 (2.7)	7 (5.7)	
500 mL	0 (0.0)	0 (0.0)		6 (0.8)	10 (1.7)		6 (1.5)	6 (2.3)		1 (0.4)	5 (4.0)	
600 mL	0 (0.0)	0 (0.0)		3 (0.4)	1 (0.2)		1 (0.2)	2 (0.8)		0 (0.0)	0 (0.0)	
>700 mL or 0 mL	1 (1.4)	0 (0.0)		7 (1.0)	5 (0.9)		4 (1.0)	1 (0.4)		0 (0.0)	1 (0.8)	
**Delivery frequency** [*N* ^l^ (%)]												
Never/Rarely	36 (50.7)	7 (10.9)	**<0.001**	326 (44.3)	106 (18.2)	**<0.001**	166 (40.6)	44 (16.9)	**<0.001**	93 (40.8)	25 (19.7)	**<0.001**
1–3 times per month	21 (29.6)	27 (42.2)		265 (36.1)	222 (38.2)		145 (35.4)	96 (36.8)		75 (32.9)	36 (28.4)	
1–2 times per week	11 (15.5)	22 (34.4)		122 (16.6)	181 (31.2)		87 (21.3)	93 (35.6)		48 (21.0)	46 (36.2)	
3–6 times per week	3 (4.2)	4 (6.3)		16 (2.2)	59 (10.2)		6 (1.5)	24 (9.2)		12 (5.3)	16 (12.6)	
1 time per day	0 (0.0)	2 (3.1)		4 (0.5)	10 (1.7)		4 (1.0)	3 (1.1)		0 (0.0)	4 (3.1)	
2 times per day	0 (0.0)	2 (3.1)		2 (0.3)	3 (0.5)		1 (0.2)	1 (0.4)		0 (0.0)	0 (0.0)	
**Coffees per day** [*N* ^m^ (%)]												
0	17 (23.9)	21 (32.8)	0.548	125 (17.0)	102 (17.5)	0.345	44 (10.7)	30 (11.5)	0.460	22 (9.6)	15 (11.8)	0.565
1	19 (26.8)	18 (28.1)		197 (26.8)	157 (27.0)		87 (21.2)	49 (18.8)		43 (18.9)	33 (26.0)	
2	22 (31.0)	19 (29.7)		237 (32.2)	209 (36.0)		144 (35.1)	86 (33.0)		82 (36.0)	37 (29.1)	
3	8 (11.3)	5 (7.8)		118 (16.0)	71 (12.2)		85 (20.7)	71 (27.2)		46 (20.2)	25 (19.7)	
4	2 (2.8)	0 (0.0)		37 (5.0)	23 (4.0)		31 (7.6)	16 (6.1)		19 (8.3)	8 (6.3)	
More than 4	3 (4.2)	1 (0.6)		22 (3.0)	19 (3.3)		19 (4.7)	9 (3.4)		16 (7.0)	9 (7.1)	
**Type of milk for coffee** [*N* ^n^ (%)]												
No fat (0%)	2 (3.0)	3 (6.3)	0.130	40 (6.4)	46 (9.6)	**<0.001**	30 (7.9)	19 (8.3)	**<0.001**	22 (10.1)	10 (8.7)	**0.010**
Low fat (1.5%)	30 (44.8)	12 (25.0)		243 (38.9)	146 (30.4)		138 (36.3)	59 (25.8)		94 (42.9)	34 (29.5)	
Full fat	11 (16.4)	5 (10.4)		43 (6.9)	31 (6.5)		12 (3.2)	23 (10.0)		6 (2.7)	11 (9.6)	
No lactose	6 (9.0)	14 (29.2)		54 (8.6)	55 (11.5)		36 (9.5)	25 (10.9)		15 (6.8)	7 (6.1)	
Coconut	4 (6.0)	5 (10.4)		43 (6.9)	11 (2.3)		27 (7.1)	3 (1.3)		8 (3.7)	1 (0.9)	
Almond	3 (4.5)	2 (4.2)		28 (4.5)	17 (3.5)		11 (2.9)	5 (2.2)		7 (3.2)	5 (4.3)	
Soya	2 (3.0)	1 (2.1)		13 (2.1)	12 (2.5)		3 (0.8)	2 (0.9)		5 (2.3)	0 (0.0)	
Other ^o^	1 (1.4)	0 (0.0)		15 (2.3)	4 (0.8)		5 (1.2)	2 (0.9)		2 (0.9)	1 (0.9)	
No milk	8 (11.9)	6 (12.5)		146 (23.4)	158 (32.9)		118 (31.1)	91 (39.7)		60 (27.4)	46 (40.0)	
**Type of sugar for coffee** [*N* ^p^ (%)]												
White	25 (37.3)	15 (68.2)	**0.001**	170 (27.2)	124 (53.2)	**<0.001**	74 (19.5)	60 (58.3)	**<0.001**	49 (22.5)	25 (49.0)	**<0.001**
Brown	8 (11.9)	6 (27.3)		63 (10.1)	47 (20.2)		22 (5.8)	13 (12.6)		9 (4.1)	6 (11.8)	
Stevia	3 (4.5)	1 (4.5)		37 (6.0)	13 (5.6)		33 (8.7)	11 (10.7)		25 (11.5)	6 (11.8)	
Honey	0 (0.0)	0 (0.0)		10 (1.6)	3 (1.3)		2 (0.5)	0 (0.0)		3 (1.4)	0 (0.0)	
Sweetener	0 (0.0)	0 (0.0)		5 (0.8)	24 (10.3)		4 (1.0)	13 (12.6)		2 (0.9)	7 (13.7)	
No sugar	31 (46.3)	0 (0.0)		339 (54.3)	22 (9.4)		245 (64.5)	6 (5.8)		130 (59.6)	7 (13.7)	

Abbreviations: BMI= Body Mass Index. During and after lockdown respectively: ^a^ *N* = 1446 and 1033; ^b^ *N* = 1443 and 1035; ^c^ *N* = 1443 and 1036; ^d^ *N* = 1446 and 1033; ^e^ *N* = 1445 and 1032; ^f^ N = 1445 and 1035; ^g^ *N* = 1440 and 1027; ^h^ *N* = 1438 and 1025; ^i^ *N* = 1441 and 1030; ^j^ *N* = 1445 and 1031; ^k^ *N* = 1424 and 1020; ^l^ *N* = 1443 and 1033; ^m^ *N* = 1445 and 1033; ^n^ *N* = 1239 and 848; ^o^ Other types of milk included evaporate, rice and goat; ^p^ *N* = 1237 and 401; ^q^ Differences in dietary habits among the first cross-sectional study and second cross-sectional study were tested using chi^2^ test. Bold values indicate statistically significant associations (*p* < 0.05).

## Data Availability

The datasets used and/or analyzed during the current study are available from the corresponding author on reasonable request.

## Supplementary Materials

The following supporting information can be downloaded at: https://www.mdpi.com/article/10.3390/foods11141994/s1, Table S1: Olive oil consumption and alcohol intake among the first cross-sectional survey (during lockdown) and the second cross-sectional survey (post-lockdown) and for females and males separately; Table S2: Delivery frequency and coffee consumption among the first cross-sectional survey (during lockdown) and the second cross-sectional survey (post-lockdown) and for females and males separately; Table S3: Dietary habits among the first cross-sectional survey (during lockdown) and the second cross-sectional survey (post-lockdown) among physical activity categories.
