# Supplementary material for: Change of Dietary and Lifestyle Habits during and after the COVID-19 Lockdown in Cyprus: An Analysis of Two Observational Studies
Source: Foods. 2022 Jul 6;11(14):1994. doi: 10.3390/foods11141994 (PMC9319624; doi:10.3390/foods11141994)
Supplement: Supplementary file 1 [file foods-11-01994-s001.zip › foods-1778352-supplementary.pdf]

Supplementary Table S1. Olive oil consumption and alcohol intake among the first cross-sectional survey (during lockdown) and the second cross-sectional survey (post-lockdown) and for females and males separately.

	**Cross-sectional study**			**During lockdown** (*N* = 1460)			**Post-lockdown** (*N* = 1043)		
**Dietary habits**	**During lockdown** (*N* = 1460, 58.3%)	**Post-lockdown** (*N* = 1043, 41.7%)	***p*-value** [c]	**Female** (*N* = 875, 59.9%)	**Male** (*N* = 57, 40.1%)	***p*-value** [d]	**Female** (*N* = 719, 68.9%)	**Male** (*N* = 324, 31.1%)	***p*-value** [d]
**Olive oil** [*N* [a] (%)]									
Never	18 (1.2)	14 (1.3)	**<0.001**	13 (72.2)	5 (27.8)	**0.009**	10 (71.4)	4 (28.6)	**0.001**
Rarely	96 (6.6)	119 (11.5)		67 (69.8)	29 (30.2)		91 (76.5)	28 (23.5)	
Less than 1 portion/week	71 (4.9)	67 (6.5)		41 (58.6)	29 (41.4)		44 (65.7)	23 (34.3)	
1-3 portions/week	328 (22.5)	299 (28.8)		190 (58.6)	134 (41.4)		185 (61.9)	114 (38.1)	
3-5 portions/week	333 (22.8)	226 (21.8)		176 (53.3)	154 (46.7)		146 (64.6)	80 (35.4)	
Daily	612 (42.0)	313 (30.1)		388 (64.0)	218 (36.0)		239 (76.4)	74 (23.6)	
**Alcohol intake** [*N* [b] (%)]									
<300 ml	1309 (91.1)	891 (86.8)	**0.012**	833 (64.2)	464 (35.8)	**<0.001**	651 (73.1)	240 (26.9)	**<0.001**
300 ml	72 (5.0)	75 (7.3)		16 (22.2)	56 (77.8)		31 (41.3)	44 (58.7)	
400 ml	27 (1.9)	29 (2.8)		5 (18.5)	22 (81.5)		13 (44.8)	16 (55.2)	
500 ml	13 (0.9)	21 (2.0)		2 (16.7)	10 (83.3)		7 (33.3)	14 (66.7)	
600 ml	4 (0.3)	3 (0.3)		0 (0.0)	4 (100.0)		1 (33.3)	2 (66.7)	
>700 ml or 0 ml	12 (0.8)	8 (0.8)		3 (27.3)	8 (72.7)		2 (25.0)	6 (75.0)	

During and after lockdown respectively: [a] *N* = 1458 and 1038; [b] *N* = 1437 and 1027; [c] Differences between dietary habits among the first cross-sectional study and the second cross-sectional study were tested using chi2 test; [d] Differences between dietary habits of females and males were tested using chi2 test; Bold values indicate statistically significant associations ($p < 0.05$).

Supplementary Table S2. Delivery frequency and coffee consumption among the first cross-sectional survey (during lockdown) and the second cross-sectional survey (post-lockdown) and for females and males separately.

	**Cross-sectional study**			**During lockdown** (*N* = 1460)			**Post-lockdown** (*N* = 1043)		
**Dietary habits**	**During lockdown** (*N* = 1460, 58.3%)	**Post-lockdown** (*N* = 1043, 41.7%)	***p*-value** [f]	**Female** (*N* = 875, 59.9%)	**Male** (*N* = 57, 40.1%)	***p*-value** [g]	**Female** (*N* = 719, 68.9%)	**Male** (*N* = 324, 31.1%)	***p*-value** [g]
**Delivery frequency** [*N* [a] (%)]									
Never/Rarely	626 (43.0)	182 (17.5)	**<0.001**	418 (67.5)	201 (32.5)	**<0.001**	126 (69.2)	56 (30.8)	**<0.001**
1-3 times per month	513 (35.2)	385 (37.0)		291 (57.1)	219 (42.9)		278 (72.2)	107 (27.8)	
1-2 times per week	269 (18.5)	343 (33.0)		147 (55.1)	120 (44.9)		240 (70.0)	103 (30.0)	
3-6 times per week	37 (2.5)	105 (10.1)		14 (37.8)	23 (62.2)		58 (55.2)	47 (44.8)	
1 time per day	9 (0.6)	19 (1.8)		3 (37.5)	5 (62.5)		11 (57.9)	8 (42.1)	
2 times per day	3 (0.2)	6 (0.6)		1 (33.3)	2 (66.7)		3 (50.0)	3 (50.0)	
**Coffees per day** [*N* [b] (%)]									
0	209 (14.3)	169 (16.3)	0.411	136 (65.7)	71 (34.3)	**0.013**	119 (70.4)	50 (29.6)	**<0.001**
1	352 (24.1)	259 (24.9)		222 (63.1)	130 (36.9)		197 (76.1)	62 (23.9)	
2	489 (33.5)	353 (33.9)		301 (61.9)	185 (38.1)		256 (72.5)	97 (27.5)	
3	259 (17.8)	172 (16.5)		144 (56.7)	110 (43.3)		99 (57.6)	73 (42.4)	
4	90 (6.2)	48 (4.6)		46 (52.3)	42 (47.7)		28 (58.3)	20 (41.7)	
More than 4	60 (4.1)	39 (3.8)		26 (44.1)	33 (55.9)		18 (46.2)	21 (53.8)	
**Type of milk for coffee** [*N* [c] (%)]									
No fat (0%)	94 (7.3)	78 (9.2)	**<0.001**	57 (62.0)	35 (38.0)	**<0.001**	67 (85.9)	11 (14.1)	**<0.001**
Low fat (1.5%)	478 (38.2)	242 (28.3)		322 (68.1)	151 (31.9)		190 (78.5)	52 (21.5)	
Full fat	69 (5.5)	67 (7.8)		32 (47.1)	36 (52.9)		41 (61.2)	26 (38.8)	
No lactose	111 (8.8)	101 (11.8)		77 (69.4)	34 (30.6)		88 (87.1)	13 (12.9)	
Coconut	82 (6.4)	25 (2.9)		52 (64.2)	29 (35.8)		22 (91.7)	2 (8.3)	
Almond	46 (3.7)	24 (2.8)		36 (78.3)	10 (21.7)		25 (100.0)	0 (0.0)	

Soya	20 (1.6)	15 (1.8)		17 (85.0)	3 (15.0)		14 (93.3)	1 (6.7)	
Other [d]	23 (1.8)	7 (0.8)		19 (82.6)	4 (17.4)		5 (71.4)	2 (28.6)	
No milk	329 (26.7)	295 (34.5)		129 (39.5)	198 (60.5)		144 (48.8)	151 (51.2)	
**Type of sugar for coffee** [*N* [e] (%)]									
White	300 (24.0)	218 (54.1)	**<0.001**	169 (56.9)	128 (43.1)	0.050	138 (63.3)	80 (36.7)	0.169
Black	102 (8.2)	72 (17.9)		54 (52.9)	48 (47.1)		51 (70.8)	21 (29.2)	
Stevia	98 (7.8)	31 (7.7)		68 (70.1)	29 (29.9)		24 (77.4)	7 (22.6)	
Honey	15 (1.2)	3 (0.7)		9 (60.0)	6 (40.0)		1 (33.3)	2 (66.7)	
Sweetener	11 (0.9)	44 (10.9)		9 (90.0)	1 (10.0)		34 (77.3)	10 (22.7)	
No sugar	724 (57.9)	35 (8.7)		430 (59.9)	288 (40.1)		26 (74.3)	9 (25.7)	

During and after lockdown respectively: [a] *N* = 1457 and 1040; [b] *N* = 1459 and 1040; [c] *N* = 1252 and 854; [d] Other types of milk included evaporate, rice and goat; [e] *N* = 1250 and 403; [f] Differences between dietary habits among the first cross-sectional study and the second cross-sectional study were tested using chi2 test; [g] Differences between dietary habits of females and males were tested using chi^2 test; Bold values indicate statistically significant associations ($p < 0.05$).

Supplementary Table S3. Dietary habits among the first cross-sectional survey (during lockdown) and the second cross-sectional survey (post-lockdown) among physical activity categories.

	**During lockdown** (*N* = 1460)			**Post-lockdown** (*N* = 1043)		
**Dietary habits**	**Not adequately physical active** (*N* = 496, 34.1%)	**Physical active** (*N* = 959, 65.9%)	***p*-value** [q]	**Not adequately physical active** (*N* = 463, 44.5%)	**Physical active** (*N* = 578, 55.5%)	***p*-value** [q]
**Non-refined cereals** [*N* [a] (%)]						
Never	124 (25.0)	127 (13.2)	**<0.001**	125 (27.2)	71 (12.3)	**<0.001**
1-6 portions/week	253 (51.0)	480 (50.1)		219 (47.6)	302 (52.3)	
7-12 portions/week	71 (14.3)	221 (23.0)		70 (15.2)	106 (18.3)	
13-18 portions/week	32 (6.5)	77 (8.0)		24 (5.2)	61 (10.5)	
19-31 portions/week	15 (3.0)	40 (4.2)		16 (3.5)	25 (4.3)	
>32 portions/week	1 (0.2)	14 (1.5)		6 (1.3)	13 (2.3)	
**Fruits** [*N* [b] (%)]						
Never	24 (4.9)	38 (4.0)	**<0.001**	44 (9.5)	23 (4.0)	**<0.001**
1-4 portions/week	204 (41.3)	261 (27.2)		192 (41.5)	174 (30.1)	
5-8 portions/week	137 (27.7)	256 (26.7)		115 (24.8)	155 (26.9)	
9-15 portions/week	81 (16.4)	228 (23.8)		59 (12.7)	125 (21.7)	
16-21 portions/week	28 (5.7)	101 (10.5)		29 (6.3)	58 (10.0)	
>22 portions/week	20 (4.0)	74 (7.8)		24 (5.2)	42 (7.3)	
**Vegetables** [*N* [c] (%)]						
Never	18 (3.6)	21 (2.2)	**<0.001**	16 (3.5)	14 (2.4)	**0.017**
1-6 portions/week	224 (45.3)	283 (29.5)		196 (42.3)	186 (32.2)	
7-12 portions/week	147 (29.8)	317 (33.1)		117 (25.3)	169 (29.2)	
13-20 portions/week	60 (12.2)	209 (21.8)		63 (13.6)	94 (16.3)	
21-32 portions/week	30 (6.1)	90 (9.4)		44 (9.5)	71 (12.3)	
>33 portions/week	15 (3.0)	38 (4.0)		27 (5.8)	44 (7.6)	
**Legumes/pulses** [*N* [d] (%)]						
Never	24 (4.8)	52 (5.3)	0.158	60 (13.0)	41 (7.1)	**0.001**

Less than 1 portion/week	89 (17.9)	128 (13.5)		109 (23.6)	113 (19.7)	
1-2 portions/week	250 (50.5)	475 (49.5)		217 (47.0)	291 (50.6)	
3-4 portions/week	116 (23.4)	262 (27.3)		61 (13.2)	109 (19.0)	
5-6 portions/week	12 (2.4)	33 (3.5)		14 (3.0)	14 (2.4)	
>6 portions/week	5 (1.0)	9 (0.9)		1 (0.2)	7 (1.2)	
**Potatoes** [*N* [e] (%)]						
Never	18 (3.6)	66 (6.9)	**0.005**	12 (2.6)	55 (9.5)	**<0.001**
1-4 portions/week	368 (74.2)	728 (76.0)		288 (62.6)	372 (64.5)	
5-8 portions/week	68 (13.7)	115 (12.0)		74 (16.1)	84 (14.6)	
9-12 portions/week	24 (4.9)	37 (3.9)		46 (10.0)	32 (5.5)	
13-18 portions/week	13 (2.6)	9 (0.9)		26 (5.7)	25 (4.3)	
>18 portions/week	5 (1.0)	3 (0.3)		14 (3.0)	9 (1.6)	
**Fish** [*N* [f] (%)]						
Never	55 (11.1)	85 (8.9)	0.120	85 (18.3)	64 (11.1)	**<0.001**
Less than 1 portion/week	174 (35.1)	288 (30.0)		193 (41.7)	204 (35.4)	
1-2 portions/week	211 (42.5)	458 (47.8)		137 (29.6)	228 (39.5)	
3-4 portions/week	44 (8.9)	108 (11.3)		41 (8.9)	63 (10.9)	
5-6 portions/week	9 (1.8)	16 (1.7)		7 (1.5)	15 (2.6)	
>6 portions/week	3 (0.6)	3 (0.3)		0 (0.0)	3 (0.5)	
**Meat and meat products** [*N* [g] (%)]						
1 or less than 1 portion/week	213 (43.3)	457 (47.8)	0.085	198 (43.2)	278 (48.4)	0.105
2-3 portions/week	152 (30.9)	316 (33.0)		137 (29.9)	174 (30.3)	
4-5 portions/week	79 (16.0)	106 (11.1)		69 (15.1)	68 (11.9)	
6-7 portions/week	27 (5.5)	45 (4.7)		26 (5.7)	35 (6.1)	
8-10 portions/week	17 (3.5)	24 (2.5)		18 (3.9)	15 (2.6)	
>10 portions/week	4 (0.8)	9 (0.9)		10 (2.2)	4 (0.7)	
**Poultry** [*N* [h] (%)]						
3 or less than 3 portions/week	291 (59.3)	518 (54.2)	0.180	199 (43.5)	269 (46.9)	**0.048**
4-5 portions/week	113 (23.0)	239 (25.0)		96 (21.0)	145 (25.3)	

5-6 portions/week	38 (7.7)	96 (10.0)		62 (13.6)	57 (9.9)	
7-8 portions/week	30 (6.1)	61 (6.4)		43 (9.4)	56 (9.8)	
9-10 portions/week	16 (3.3)	25 (2.6)		40 (8.8)	29 (5.1)	
>10 portions/week	3 (0.6)	17 (1.8)		17 (3.7)	17 (3.0)	
**Full-fat dairy products** [*N* [i] (%)]						
10 or less than 10 portions/week	328 (66.5)	663 (69.3)	0.365	272 (59.1)	395 (68.7)	**0.001**
11-15 portions/week	94 (19.1)	157 (16.4)		84 (18.2)	91 (15.8)	
16-20 portions/week	31 (6.3)	76 (7.9)		32 (7.0)	44 (7.6)	
21-28 portions/week	23 (4.7)	31 (3.2)		30 (6.5)	24 (4.2)	
29-30 portions/week	10 (2.0)	14 (1.5)		27 (5.9)	13 (2.3)	
>30 portions/week	7 (1.4)	16 (1.7)		15 (3.3)	7 (1.4)	
**Olive oil** [*N* [j] (%)]						
Never	4 (0.8)	14 (1.5)	0.169	7 (1.5)	7 (1.2)	**0.032**
Rarely	33 (4.7)	63 (6.6)		62 (13.5)	57 (9.9)	
Less than 1 portion/week	24 (4.8)	46 (4.8)		32 (6.9)	35 (6.1)	
1-3 portions/week	131 (26.4)	196 (20.5)		148 (32.0)	151 (26.3)	
3-5 portions/week	109 (22.0)	223 (23.3)		95 (20.6)	131 (22.9)	
Daily	195 (39.3)	415 (43.3)		118 (25.5)	193 (33.6)	
**Alcohol intake** [*N* [k] (%)]						
<300 ml	443 (91.3)	861 (90.9)	0.654	377 (83.4)	512 (89.4)	**0.004**
300 ml	27 (5.6)	45 (4.8)		45 (10.0)	30 (5.2)	
400 ml	7 (1.5)	20 (2.1)		14 (3.1)	15 (2.6)	
500 ml	4 (0.8)	9 (1.0)		12 (2.6)	9 (1.6)	
600 ml	0 (0.0)	4 (0.4)		3 (0.7)	0 (0.0)	
>700 ml or 0 ml	4 (0.8)	8 (0.8)		1 (0.2)	7 (1.2)	
**Delivery frequency** [*N* [l] (%)]						
Never/Rarely	208 (42.1)	415 (43.3)	0.890	73 (15.9)	109 (18.9)	0.170
1-3 times per month	171 (34.6)	340 (35.5)		164 (35.6)	221 (38.2)	
1-2 times per week	99 (20.1)	170 (17.8)		157 (34.1)	184 (31.8)	

3-6 times per week	13 (2.6)	24 (2.5)		50 (10.9)	55 (9.5)	
1 time per day	2 (0.4)	7 (0.7)		11 (2.4)	8 (1.4)	
2 times per day	1 (0.2)	2 (0.2)		5 (1.1)	1 (0.2)	
**Coffees per day** [*N*[m] (%)]						
0	63 (12.7)	146 (15.2)	0.192	75 (16.3)	94 (16.2)	0.200
1	116 (23.4)	235 (24.5)		102 (22.2)	156 (27.0)	
2	176 (35.5)	310 (32.4)		152 (33.0)	201 (34.8)	
3	80 (16.1)	178 (18.6)		85 (18.5)	86 (14.9)	
4	34 (6.9)	56 (5.9)		26 (5.7)	22 (3.8)	
More than 4	27 (5.4)	33 (3.4)		20 (4.3)	19 (3.3)	
**Type of milk** [*N* [n] (%)]						
No fat (0%)	29 (6.7)	65 (8.0)	0.292	39 (10.3)	39 (8.2)	**0.013**
Low fat (1.5%)	184 (42.3)	293 (36.1)		108 (28.6)	133 (28.0)	
Full fat	22 (5.1)	46 (5.7)		44 (11.7)	23 (4.8)	
No lactose	44 (10.1)	66 (8.1)		39 (10.3)	62 (13.0)	
Coconut	24 (5.5)	57 (7.0)		7 (1.9)	17 (3.6)	
Almond	12 (2.8)	34 (4.1)		8 (2.1)	17 (3.6)	
Soya	8 (1.8)	12 (1.5)		5 (1.3)	10 (2.1)	
Other[o]	11 (2.5)	12 (1.5)		1 (0.4)	6 (1.3)	
No milk	101 (23.2)	227 (28.0)		126 (33.4)	168 (35.4)	
**Type of sugar** [*N* [p] (%)]						
White	121 (28.0)	178 (21.9)	0.099	118 (62.8)	99 (46.3)	**0.003**
Brown	29 (6.7)	72 (8.9)		27 (14.4)	45 (21.0)	
Stevia	38 (8.8)	60 (7.4)		11 (5.8)	20 (9.3)	
Honey	6 (1.4)	9 (1.1)		2 (1.1)	22 (10.3)	
Sweetener	5 (1.2)	6 (0.7)		22 (11.7)	1 (0.5)	
No sugar	233 (53.9)	488 (60.0)		8 (4.2)	27 (12.6)	

During and after lockdown respectively: [a] *N* = 1455 and 1038; [b] *N* = 1452 and 1040; [c] *N* = 1452 and 1041; [d] *N* = 1455 and 1037; [e] *N* = 1454 and 1037; [f] *N* = 1454 and 1040; [g] *N* = 1449 and 1032; [h] *N* = 1447 and 1030; [I] *N* = 1450 and 1034; [j] *N* = 1453 and 1036; [k] *N* = 1432 and 1025; [l] *N* = 1452 and 1038; [m] *N* = 1454 and 1038; [n] *N* = 1247 and 852; [o] Other types of milk

included evaporate, rice and goat; [p] N = 1245 and 402; [q] Differences between dietary habits among physical activity groups were tested using chi2 test; Bold values indicate statistically significant associations (p<0.05).
